# Dietary Implications of the Bidirectional Relationship between the Gut Microflora and Inflammatory Diseases with Special Emphasis on Irritable Bowel Disease: Current and Future Perspective

**DOI:** 10.3390/nu15132956

**Published:** 2023-06-29

**Authors:** Tariq Aziz, Ayaz Ali Khan, Athina Tzora, Chrysoula (Chrysa) Voidarou, Ioannis Skoufos

**Affiliations:** 1 Laboratory of Animal Health, Food Hygiene and Quality, Department of Agriculture, University of Ioannina, 47100 Arta, Greece; 2 Department of Biotechnology, University of Malakand, Chakdara 18800, Pakistan

**Keywords:** polymyalgia rheumatica (PMR), spinal muscular atrophy (SMA), vasculitis, sarcopenia, cirrhosis, cancer, fibromyalgia, psoriasis, sarcoidosis, lupus, arthritis

## Abstract

The immune system is vital for safeguarding the human body against infections and inflammatory diseases. The role of diet and meal patterns in modulating immune function is complex, and highlighting this topic is crucial for identifying potential ways to improve immune health. In Europe, the Mediterranean diet and Western diet are the most common dietary patterns, and gaining an understanding of how they affect immune function is essential for public health. There are numerous inflammatory diseases that are observed in younger and older people. Some of the common diseases include polymyalgia rheumatica (PMR), spinal muscular atrophy (SMA), vasculitis, sarcopenia, cirrhosis, cancer, and fibromyalgia, but the main focus in this review article is on irritable bowel disease (IBD). In general, dietary choices can have an immense impact on the microbial flora of the gut in people with inflammatory diseases. The intake of Mediterranean-style foods promotes the growth of healthy bacteria that enhances the function of the immune system. On the other hand, it is mostly seen that the intake of Western-style foods leads to the growth of harmful gut bacteria that contributes to inflammation and disease development by weakening the immune system. Additionally, inflammation in the gut can impact brain function, leading to mood disorders, such as anxiety and depression. Rare inflammatory diseases, such as psoriasis and sarcoidosis, are of main interest in this article. All the above-mentioned common and rare inflammatory diseases have a certain relationship with the microbiota of the gut. The gut microbiome plays a significant role in IBD; fiber and prebiotic interventions may represent promising adjunct therapies for pediatric IBD by targeting the gut microbiome. By advancing a good overall arrangement of microorganisms in the stomach through dietary mediations, working on the side effects and alleviating of diseases might be conceivable. The gut microbiota can be affected differently by various dietary fatty acid types. There is also an involvement of genetics in the progression of IBD, such as transcriptional factors, and one gene of interest is the *LCT* gene, which encodes for lactase, an enzyme responsible for digesting lactose in the gut.

## 1. Introduction

One of the most vital elements which has an immense impact on microbial composition is the diet, which is further linked to the morpho-functional integrity of the intestinal barrier and host immunity [1]. Fluctuating the intake and specific food groups would promote gut dysbiosis, which leads to an alteration of the gut barrier, immune activation, and tissue damage [2]. Moreover, it can also have a role in the development of irritable bowel disease (IBD) and other inflammatory diseases. The Western diet, which is high in saturated fat, red meat, and refined sugars, and is low in fiber, fresh fruits, and vegetables, is considered a possible risk factor in the development of IBD. Considering complex diseases where diet is a contributing factor, it is hard to assess the role of any single food because dietary patterns involve exposure to various groups of foods. Till now, evidence on how diet influences IBD and other inflammatory diseases is insufficient [3]. In addition to that, the impact the food choices may have on the disease courses remains unknown apart from exclusive or partial enteral nutrition, which has been shown to induce remission in patients affected by Crohn’s disease (CD) [4,5].

**Figure 1:**
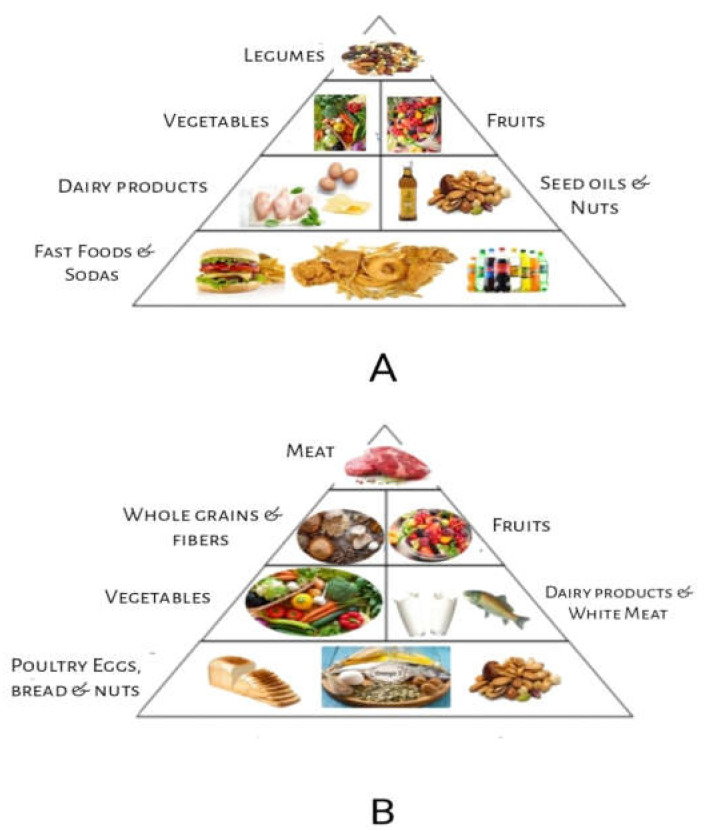
Food Pyramids: (**A**) Western food style pyramid; (**B**) Mediterranean food style pyramid.

**Figure 2:**
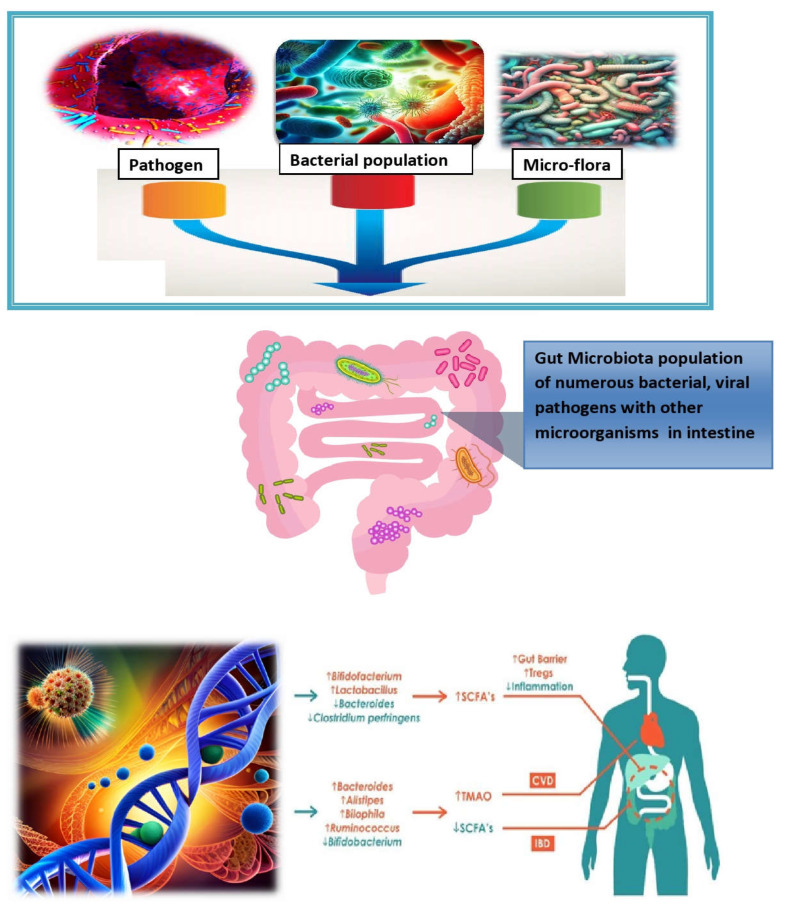
Exploring the multifactorial roots of microbiota-associated inflammation.

The health of the immune system and the body’s immunological response are significantly influenced by the gut microbiota, which is made up of billions of bacteria that live in the gastrointestinal tract. Recent research reveals that dietary variables, among others, can have a major impact on the makeup and operation of the gut microbiota [6]. According to research, the gut microbiota has a significant influence on immune function and the body’s capacity to fight off illnesses and infections. An immune system can be supported by a nutritious, well-balanced diet that is low in substances that cause inflammation. However, the link between nutrition and immunological health is complicated, with genetics and way of life both having a big impact [7]. The Mediterranean diet and the Western diet are two dietary styles that are popular among Europeans. Lower levels of inflammation and a decreased risk of such chronic illnesses as cancer and cardiovascular disease have been linked to the Mediterranean diet (Figure 1), which places emphasis on whole grains, fruits, vegetables, and healthy fats [8]. The Western diet (Figure 2), on the other hand, has been associated with increased inflammation and a higher risk of chronic illnesses since it is heavy in processed foods, red and processed meat, and refined carbohydrates. Saturated and trans-fat-rich diets, common in Western diets, might encourage inflammation and decrease immunological function. Additionally, consuming too much sugar can cause chronic inflammation and increase the chance of developing autoimmune illnesses [9].

Saturated and trans-fat-rich diets are common in Western diets compared to the Mediterranean diet.

The immune system’s performance can be impacted by eating habits. Animal studies have demonstrated that intermittent fasting, which involves limiting food intake for a set period, enhances immune function. Intermittent fasting has been linked to lowered inflammatory markers and enhanced immunological function in people [10]. Unbalanced gut microbiota has been related to inflammatory illnesses, including inflammatory bowel disease (IBD). A growing body of research indicates that nutrition, through altering the makeup and functionality of the gut microbial flora, plays a substantial role in the development and progression of many disorders [11]. Studies have shown that diets high in refined carbohydrates and saturated fats might increase potentially hazardous bacteria while reducing the number of good bacteria in the gut. On the other hand, it has been found that diets high in fiber, vegetables, fruits, and whole grains encourage the growth of healthy bacteria and lessen gastrointestinal inflammation [12].

Prebiotics and probiotics, two dietary components, have regulatory effects on individuals with inflammatory disorders. Non-digestible fibers called prebiotics promote the development and activity of good bacteria in the gut. By introducing healthy living bacteria into the stomach, probiotics can enhance health by being eaten in appropriate proportions [13]. In general, food can have a significant effect on the microbial flora of the stomach in people with inflammatory illnesses. It could be feasible to lessen the course of certain illnesses and reduce symptoms by encouraging healthy and varied gut microbiota through dietary treatments. It is possible to control the signs and symptoms of these disorders, as well as lessen their consequences by altering the diet to create the ideal bacteria environment in the gut. Inflammatory diseases, such as PMR, SMA, vasculitis, sarcopenia, cirrhosis, cancer, and fibromyalgia, are discussed in this review article.

Inflammatory diseases, such as irritable bowel disease (IBD), are chronic inflammatory conditions associated with an imbalance in the gut microflora. There is expanding proof to suggest that dietary factors play a significant role in the disease onset and progression by affecting the gut microbial flora composition and its function [14]. For example, several experiments have revealed that diets rich in saturated fats and refined carbohydrates can cause a decrease in the growth of normal intestinal flora while increasing the abundance of potentially harmful bacteria. In contrast, however, a diet comprising fibers, vegetables, fruits, whole grains, etc., has been observed to increase the healthy and beneficial bacteria’s growth and reduce inflammation in the gut [15]. Certain dietary components, such as prebiotics and probiotics, also have regulatory effects in patients suffering from inflammatory diseases. Prebiotics, which are non-digestible fibers, enhance the multiplication and functions of normal flora in the gut, while probiotics, when taken in enough amounts, also provide health benefits [16]. Fiber and prebiotic interventions have emerged as potential therapeutic strategies for IBD, as they can modulate the gut microbiome and promote the growth of beneficial bacteria. Several studies have demonstrated the efficacy of these interventions in improving clinical outcomes and reducing disease activity in pediatric patients with IBD [17]. For instance, a randomized controlled trial showed that a high-fiber diet led to a significant reduction in disease activity in pediatric patients with Crohn’s disease. Similarly, a meta-analysis of randomized controlled trials found that prebiotic interventions improved symptoms and reduced inflammation in patients with ulcerative colitis [18].

The microgram population affects the digestive gut of higher animals by producing its colonies in numbers which eventually damage the functional structure of the gut. This leads to a complex interplay of factors influencing microbiota-associated chronic inflammation in health, such as uncovering the complex web of factors involved in microbiota-associated chronic inflammation and mapping the landscape of chronic inflammation in the microbiota era [19]. From lifestyle to genetics, the diverse factors impacting microbiota-driven inflammation are discussed in Figure 2. The gut microbiota has the capability to utilize several metabolic pathways, including those for trimethylamine *N*-oxide, short-chain fatty acids, and primary and secondary bile acids. By influencing these biological processes, the gut microbiota has been implicated in the pathogenesis of IBD and various cardiovascular disorders (CVD) [20]. Inflammatory diseases, such as PMR, SMA, vasculitis, sarcopenia, cirrhosis, cancer, and fibromyalgia, are discussed in this review article with special emphasis on IBD and its future perspectives.

### 1.1. Healthy Bacteria of the Gut

**Figure 3:**
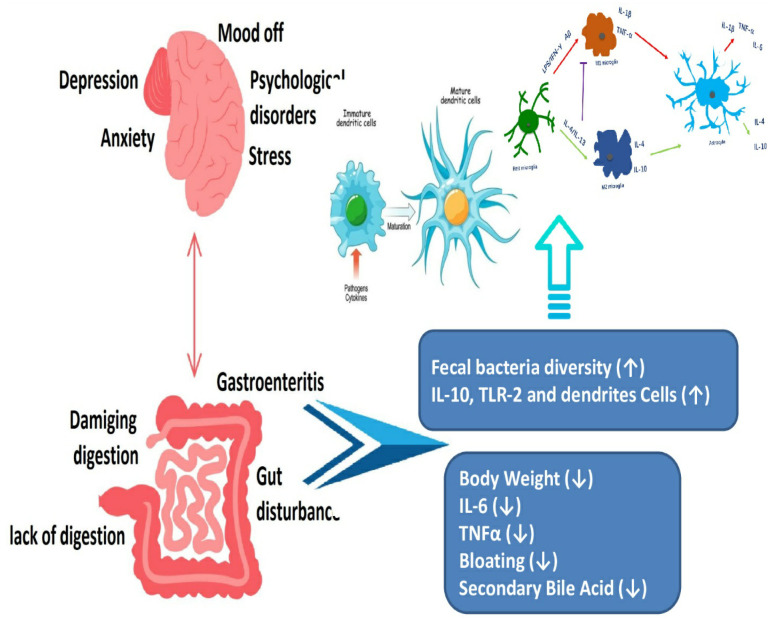
Psychological conditions can disrupt normal gastrointestinal tract function ([33] Brzozowski et al., 2016).

*Faecalibacterium prausnitzii*, *Akkermansia muciniphila*, and *Bifidobacterium* are healthy bacteria that maintain gut barrier function and regulation of immune function in the gut. *Bifidobacterium*: *Bifidobacteria* are a group of beneficial bacteria that have several benefits regarding the health of the individual. These benefits may include promoting digestive health, reduction in inflammation, and boosting the immune system [21]. *Lactobacillus* is a type of bacteria that is commonly found in the gut and has been observed to have a positive effect on the health of the gut. Therefore, it has been linked with improving the gut barrier’s function, reduction in inflammation, and promoting overall digestive health [22]. *Faecalibacterium prausnitzii* is a beneficial bacterium that has anti-inflammatory properties and may help in the prevention and treatment of IBD [23]. *Akkermansia muciniphila* is a type of bacteria that is thought to be important for the health of the gut. It also has been linked with the reduction in inflammation, improvement in the functioning of the gut barrier, and protection against metabolic disorders [24]. *Prevotella, Bacteroides*, and *Ruminococcus* species can cause inflammation and add up to the disease development by affecting the working of the immune system [25]. There is a relationship between the brain, mental health, and the digestive system (Figure 3). The gut–brain axis (GBA) is a communication system between the central nervous system and the gut. There is increasing evidence that alterations in the GBA play a role in the pathogenesis of inflammatory bowel disease (IBD) [26]. Studies have shown that stress and other psychological factors can exacerbate symptoms in patients with IBD. This bidirectional relationship between the gut and brain has led to the investigation of therapies that target the GBA, including cognitive behavioral therapy, mindfulness-based interventions, and gut-directed hypnotherapy [27].

Furthermore, the gut microflora plays a key role in GBA signaling. Dysbiosis, or an imbalance in the beneficial intestinal microbes, has been associated with alterations in GBA signaling and an increased risk of IBD. Therefore, interventions that aim to restore a healthy gut microbiome, such as probiotics, prebiotics, and fecal microbiota transplantation, may also have a beneficial effect on the GBA and improve outcomes in patients with IBD [28]. Overall, research into the GBA in IBD is a rapidly evolving field with promising avenues for future treatment development. By better understanding the complex interactions between the gut and brain in IBD, one can identify novel targets for therapy and improve outcomes for patients [29].

Growing evidence suggests that the gut microbiome plays a key role in the pathogenesis of irritable bowel syndrome (IBS) and that diet and nutrition can modulate the composition and function of the microbiome [30]. The gut microbiome produces a variety of metabolites, including amino acids, biogenic amines, and short-chain fatty acids (SCFAs), which can interact with host cells and influence gut physiology. Recent studies have identified several biochemical pathways that are dysregulated in IBS, including those involved in immune function, intestinal permeability, and mucosal inflammation [31]. Diet and nutrition can modulate these pathways by altering the production of gut microbial metabolites, such as SCFAs, which have anti-inflammatory and immunomodulatory effects. For instance, a high-fiber diet has been shown to increase the production of SCFAs and improve symptoms in patients with IBS. Similarly, probiotics and prebiotics have been shown to modulate the gut microbiome and improve clinical outcomes in patients with IBS. These findings suggest that dietary interventions targeting the gut microbiome may represent a promising approach for the management of IBS [32].

Research in IBD is expected to focus on several key areas, including the identification of novel biomarkers for disease diagnosis and prognosis, the development of personalized treatment strategies based on individual patient characteristics, and the exploration of non-pharmacological interventions, such as diet and exercise, for the management of IBD [33]. One promising area of research is the development of biologics, which are medications that target specific molecules involved in the immune response to treat IBD. In addition to traditional biologics, new biologics that target different pathways involved in the immune response are being developed. Other novel therapies include stem cell therapy, gene therapy, and fecal microflora transplantation, which involves the transfer of fecal material from a healthy donor to the patient’s gastrointestinal tract to restore the gut microbiome [34].

### 1.2. Fatty Acids and Gut Health

#### 1.2.1. Composition of Fatty Acids

The gut microbiota can be affected differently by various dietary fatty acid types, such as saturated fatty acids (SFAs), monounsaturated fatty acids (MUFAs), and polyunsaturated fatty acids (PUFAs). For instance, a high intake of SFAs has been linked to unfavorable alterations in the makeup of the gut microbiota, which may encourage the development of dangerous bacteria. However, some PUFAs, such as omega-3 fatty acids, have been associated with a better gut microbial composition [35].

#### 1.2.2. Composition of Gut Microbial Flora

A vital part of sustaining gut health and general well-being is the gut flora. It aids in the production of certain vitamins and short-chain fatty acids (SCFAs), as well as in digestion, nutritional absorption, immunological function, and nutrient absorption. Dysbiosis, or imbalances in the makeup of the gut microbiota, has been linked to a number of illnesses, such as inflammatory bowel disease (IBD), obesity, and metabolic abnormalities. Immune function may be impacted by changes in the makeup of the gut microbiota, which interacts with the host’s immune system. SFAs, especially those originating from animal sources, can increase inflammation and stimulate the immune system, which may cause inflammatory illnesses to manifest. In contrast, some PUFAs, such as omega-3 fatty acids, have anti-inflammatory characteristics and may contribute to a favorable modulation of immune responses. The development of different illnesses may be influenced by dysbiosis, and inflammation brought on by an unhealthy diet low in BDFAs and high in SFAs. For instance, variations in the composition and function of the gut microbiota have been associated with obesity and metabolic diseases, and dysbiosis has been implicated in the pathogenesis of IBD. Inflammation brought on by dysbiosis may potentially have an effect on cardiovascular health [36].

### 1.3. Association between Common Inflammatory Diseases and Gut Microbiota with Reference to Diet

#### 1.3.1. Polymyalgia Rheumatica (PMR)

Polymyalgia rheumatica (PMR) is one of the types of inflammatory disorders that mostly affects individuals aged over 50. The exact cause of PMR is unclear, but there is evidence that shows that dietary factors have a role in the progression and disease development. Some studies have suggested that certain dietary supplements, such as vitamin D, may be beneficial for individuals with PMR. For example, in 2015, a study found that vitamin D supplementation may reduce the need for corticosteroid therapy in patients with PMR [37].

A 2019 study found that a diet containing processed foods, red meat, and refined sugars might be associated with a higher risk of developing PMR (Zhao et al., 2019) [25]. On the other hand, a diet that includes vegetables, fruits, protein sources, and flatbread may have a protective effect against PMR. Some of the bacterial species that may be associated with this condition include *Prevotella, Bacteroides,* and *Ruminococcus,* which are drastically abundant in the gut of the patients compared to the microbial flora of the gut in normal healthy individuals [38]. Consuming a healthy diet that may include fruits, whole grains, a variety of vegetables, nuts, seeds, and legumes can increase the intake of prebiotic fibers that feed beneficial bacteria in the gut. For probiotic bacteria, such fermented foods as yogurt are good as these can also support the gut microbiota. Apart from that, avoid the intake of processed foods, added sugars, and saturated and trans fats because they may largely contribute to inflammation [39]. Thus, all of the mentioned foods can alleviate the symptoms of inflammatory disease rather than cure the disease.

#### 1.3.2. Spinal Muscular Atrophy (SMA)

Nutrition plays a significant part in the care of patients suffering from SMA. These patients face the progressive wasting of muscles, and their functional impairment has a profound and devastating influence on the outcomes of diet [40]. As many patients with this disease must face the problem of malnutrition, a special diet and dietary fiber intake become crucial to maintaining their good health conditions [41]. A study was conducted in Boston, Massachusetts, that included 60 subjects; after the uptake of a special diet and dietary for 3 years, the cases of malnutrition decreased from 65% to 27% [42].

A systemic review was conducted on 36 studies in Australia that has shown a very important role of dietary fibers in the normal growth and muscle development of SMA patients [43]. Another study was conducted on the Chinese population in 2016; it showed that lower calcium uptake and malnutrition in 84% of the subjects could flare up SMA. They concluded that a special diet and calcium uptake could improve muscle and body growth [44]. Research has shown the link between SMA and changes in the gut microbial flora with reference to diet. One study that was conducted in the SMA animal model showed that the changes in the microbial flora of the gut were associated with disease severity and progression. Specifically, the abundance of certain bacterial taxa was altered in mice with SMA compared to control mice. The authors also found that modulating the microbiota of the gut by using probiotics has improved motor function as well as extended lifespan in mice with SMA [45]. In another study, the examination of the gut microbiota was performed in some patients who suffered from SMA. The researchers found that these patients had altered microbial diversity compared to healthy controls, which might be a key factor in the pathophysiology of SMA. The gut microbiota is targeted to represent a novel therapeutic approach to the disease [46].

#### 1.3.3. Vasculitis

The involvement of gastrointestinal is very common in patients suffering from vasculitis. In chronic systemic inflammation, patients frequently experience weight loss and cachexia; therefore, the diet should be altered accordingly [47]. Another experiment was conducted involving two groups of mice based on the differences in diet. One group contained all the necessary nutrients, and the other group lacked beta-glucan. Group two, which was free from beta-glucan, was comparatively having less survival time. Moreover, the Bacteroides were present in large numbers, which caused inflammation in the microbial flora of the gut in the second group [48].

In some cross-sectional studies, researchers have found that levels of antioxidants are inversely related to the levels of inflammatory markers. A study group was designed with a restricted diet that included simple carbohydrates and fried foods and lacked fruits and vegetables. Their results showed an increased level of CRP, explaining the link between diet and disease [49]. One of the types of vasculitis is AAV (anti-neutrophil cytoplasmic antibody-associated vasculitis), in which the patient suffers from the inflammation of small blood vessels. Neutrophils are the main members in the development of disease, and their activity is strongly influenced by some metabolites produced by the fermentation of non-digestible carbohydrates by the microbial flora of the gut [50]. For the treatment of vasculitis, patients take steroids that can lead to osteoporosis. Increased calcium in the diet helps prevent it from developing. Moreover, it is recommended to consume broccoli, skimmed milk, and yogurt [51].

#### 1.3.4. Sarcopenia

Diet may play a significant role in the onset and progression of sarcopenia, according to the available evidence. It has been demonstrated that getting enough protein, especially from high-quality sources, is necessary for older adults to keep their muscles intact, as well as their function. Omega-3 fatty acids and vitamin D may also have the potential to prevent sarcopenia. For instance, a precise survey and meta-examination of 20 investigations carried out in aged people having sarcopenia discovered that protein supplementation showed improvement in maintaining muscle mass, strength, and function [52]. According to another research, an omega-3-rich diet was linked to greater muscle mass and lower levels of inflammation in older women. At last, a randomized controlled preliminary trial led to the conclusion that vitamin D supplementation kept the activity of muscles intact and helped capability in aged people with sarcopenia [53].

Moreover, in one review, it was observed that dietary supplementation with whey protein, a high-quality protein source, can improve muscle strength, mass, functioning, and physical activity in adults with sarcopenia aged over 60 [54]. Additionally, a cross-sectional study found that a higher intake of fruits and vegetables was associated with greater muscle mass and strength in older adults [55]. Furthermore, a randomized controlled trial demonstrated that a Mediterranean-style diet improved muscle strength and physical function in older adults suffering from sarcopenia [56]. Overall, these studies suggest that a balanced diet with adequate protein, fruits, and vegetables and adherence to a Mediterranean-style diet may be beneficial for preventing and managing sarcopenia in older adults.

#### 1.3.5. Cirrhosis

Cirrhosis is a chronic liver illness that is characterized by the growth of scar tissue in lieu of healthy liver tissue. Research has revealed that nutrition has a significant impact on the onset and progression of cirrhosis. For instance, a high protein diet has been linked to a higher chance of developing hepatic encephalopathy, a cirrhosis consequence that impairs brain function [57]. Conversely, a larger diet of polyunsaturated fats has been connected to a decreased risk of liver fibrosis, which is a major cause of cirrhosis, whereas a higher intake of saturated and monounsaturated fats led to an increased risk of liver fibrosis [58].

Moreover, non-alcoholic fatty liver disease (NAFLD), a frequent cause of cirrhosis, has been related to a diet rich in sugar and refined carbohydrates [59]. Additionally, cirrhosis and its side effects, such as hepatic encephalopathy and variceal hemorrhage, have been linked to reducing intakes of dietary fiber [60]. According to a study performed in 2019, a diet rich in red and processed meat led to an increased chance of liver fibrosis, which can result in cirrhosis [61]. According to a different 2020 study, people with cirrhosis may benefit from a Mediterranean-style diet that is high in fruits, vegetables, whole grains, fish, and olive oil because it enhances liver function and lowers the risk of complications [62]. These data collectively imply that dietary changes may be crucial in the prevention and treatment of cirrhosis.

#### 1.3.6. Cancer

There are some research studies that show the link between diet and cancer. According to a 2015 study, a Western-style diet was linked to a higher risk of colorectal cancer [63]. A plant-based diet lowers the risk of breast cancer [64]. Various nutrients, such as vitamin D and omega-3 fatty acids, may also act as a preventative measure against some cancers, according to other research [65].

#### 1.3.7. Fibromyalgia

Fibromyalgia is a complex chronic pain condition that affects many aspects of a person’s life, including diet and nutrition. Numerous studies imply that dietary elements may influence the onset and treatment of fibromyalgia. For instance, a low-FODMAP diet, which limits specific carbohydrate types that may cause digestive problems, may be useful in easing fibromyalgia symptoms [66]. Other studies have shown that people with fibromyalgia may also benefit from a diet high in anti-inflammatory foods, such as fruits, vegetables, whole grains, and lean protein may also be beneficial for individuals with fibromyalgia [67,68].

#### 1.3.8. Alzheimer’s Disease

Alzheimer’s disease is a degenerative neurological condition marked by memory loss and cognitive deterioration. While the precise origin of Alzheimer’s disease is unknown, evidence indicates that food and gut flora may have an impact on the onset and course of the condition. According to one study, people with Alzheimer’s disease exhibited a different gut microbiota composition from those in general, with lower concentrations of helpful bacteria and greater concentrations of possibly dangerous bacteria [69]. According to other research, alterations in the gut microbiota might cause an increase in inflammation and oxidative stress, both of which have been associated with the onset of Alzheimer’s disease [70]. In terms of food, a Mediterranean-style diet lowers the incidence of Alzheimer’s disease. This diet places an emphasis on whole grains, fruits, vegetables, and healthy fats. This is assumed to be because many of the items of this kind of diet have anti-inflammatory and antioxidant qualities. A sugary diet, refined carbohydrates, and trans fats have a higher probability of Alzheimer’s disease [70]. Overall, there is evidence to suggest that a good diet and a balanced microbial flora of the gut may be significant factors in lowering the risk of acquiring Alzheimer’s disease, even though the relationship between Alzheimer’s disease, food, and gut microbiota is currently being investigated.

#### 1.3.9. Parkinson’s Disease

A neurodegenerative ailment called Parkinson’s disease is characterized by the brain’s dopamine-producing neurons dying out. According to recent studies, the development and course of the illness may be influenced by food and gut bacteria. Studies have revealed that the gut microbiota in people with Parkinson’s disease differs from those in health, with a reduced number of helpful bacteria and a larger abundance of possibly dangerous bacteria [71]. The course of Parkinson’s disease is assumed to be aided by increased inflammation due to the alterations in gut microbiota in another research [72]. A high-fiber diet was linked to a lower incidence of Parkinson’s disease, according to research on food [73]. Fiber is considered to encourage the development of good gut flora, which may assist in lessening oxidative stress and inflammation. On the other hand, a diet with a high potency of saturated fats and processed foods is associated with a greater chance of Parkinson’s disease [74]. There is evidence to suggest that a healthy diet and a balanced gut microbiota may be significant factors in lowering the chance of acquiring Parkinson’s disease, even though the relationship between Parkinson’s disease, food, and gut microbiota is still being investigated.

#### 1.3.10. Arthritis

Joint discomfort and inflammation are often referred to as arthritis. According to research, food and gut bacteria are involved in the onset and progression of different types of arthritis. Research has revealed that the intestinal microflora of rheumatoid arthritis (RA) patients differs from that of controls, with a reduced quantity of helpful bacteria and a larger abundance of possibly dangerous bacteria [75]. The development of RA is mostly influenced by inflammation, which has been linked to alterations in gut microbiota in another research [76]. A study on food discovered that a decreased risk of having RA was linked to a Mediterranean-style diet [77]. This is assumed to be because many of the items in this kind of diet have anti-inflammatory and antioxidant qualities. As with all other inflammatory diseases, saturated fat and sugar also play an important role in the development of RA [78]. Additionally, it has been demonstrated that several foods and supplements contain anti-inflammatory characteristics, which may be helpful for treating the symptoms of arthritis. For instance, omega-3 fatty acids, which are included in fish oil, have been demonstrated to lower inflammation and may assist people with arthritis to have less pain and stiffness in their joints [79]. Overall, there is evidence to suggest that a good diet and a balanced gut microbiota may be significant factors in lowering the risk of developing arthritis and controlling its symptoms, even though the relationship between food, arthritis, and gut microbiota is currently being investigated.

#### 1.3.11. Inflammatory Bowel Diseases

**Figure 4:**
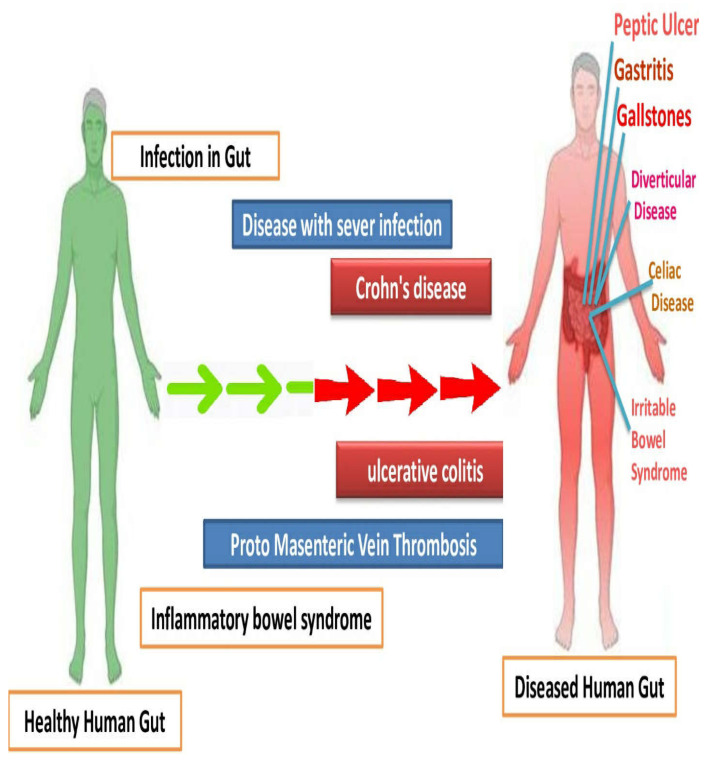
Comparison of human gut with bowel inflammatory diseases, with the control having healthy gut microbiome.

Inflammatory bowel diseases (Crohn’s disease, peptic ulcer, gastritis, ulcerative colitis diverticular disease, irritable bowel syndrome) are chronic conditions manifested by gastrointestinal tract inflammation. These diseases have a significant impact on the patients’ lifestyle quality and require long-term management. In recent years, research efforts have focused on understanding the underlying mechanisms and developing targeted therapies (Figure 4). Advances in genetic studies have identified key genetic variants associated with susceptibility to these diseases, shedding light on their complex etiology. Additionally, emerging technologies, such as microbiome analysis and precision medicine approaches, hold promise for personalized treatment strategies. Furthermore, ongoing clinical trials are exploring novel therapeutic agents targeting specific immune pathways and cytokines involved in the inflammatory process. The integration of these advancements in the diagnosis, monitoring, and treatment of IBD shows potential for improved outcomes and a brighter future for patients affected by these conditions [80,81].

Table 1 below summarizes the healthy, beneficial diet to prevent or alleviate the symptoms of the disease, and the harmful diet that triggers the disease by altering the gut microbial flora is given below.

### 1.4. Association between Rare Inflammatory Diseases and Gut Microbiota with Reference to Diet

#### 1.4.1. Sarcoidosis

The etiology of sarcoidosis, an inflammatory illness that affects several bodily organs, is yet unclear. According to recent research, dietary choices may influence the immune system and levels of oxidative stress in the body, which may contribute to the onset and progression of sarcoidosis. For example, omega-3 fatty acid-rich diets, which may be found in fish and nuts, may also be protective against sarcoidosis [82]. High consumption of fruits and vegetables strong in antioxidants has also been linked to a decreased chance of developing sarcoidosis, probably as a result of their capacity to lessen oxidative stress and inflammation in the body [83].

Conversely, Western-style food, high in trans and saturated fats, refined carbohydrates, and foods that are processed, has been associated with an increased risk of sarcoidosis [84]. Therefore, it appears that dietary factors may influence the development and progression of this disease, and adopting a healthy and balanced diet may be a potential way to manage or reduce the risk of sarcoidosis. Apart from the diet, microbial flora also plays a part in the development of disease. A study published in 2018 found that patients with sarcoidosis had increased levels of certain bacteria in their saliva in comparison to healthy controls. According to the authors’ hypothesis, these alterations in the oral microbiota may be connected to the inflammation brought on by sarcoidosis [85]. In a different study, researchers discovered that sarcoidosis patients’ gut microbiome was different from that of healthy controls. When sarcoidosis patients were compared to healthy controls, the researchers discovered substantial variations in the number of certain intestinal bacterial species. The authors hypothesized that the immunological dysregulation found in sarcoidosis may be caused by these alterations in the gut flora [86]. Overall, while there is some evidence that suggests that alterations in the microbial flora may be related to sarcoidosis, further studies are required to completely understand this association.

#### 1.4.2. Psoriasis

A persistent autoimmune condition called psoriasis causes skin inflammation and the development of scaly, red patches on the skin. Alterations in the gut microbiota may contribute to the onset and progression of psoriasis. Immune dysfunction and the formation of such autoimmune diseases as psoriasis may result from an imbalance in the gut microbiota, which is known to interact with the immune system [87]. Dietary factors have been identified as a potential modifier of the gut microbiota, with studies suggesting that a diet rich in fat and sugar may cause the number of good bacteria to decline and the number of harmful bacteria to rise. A diet high in fiber, fruits, and vegetables, on the other hand, has been linked to an increase in beneficial bacteria and a decrease in inflammation [88]. Several dietary elements, including probiotics and prebiotics, have been demonstrated in studies to alter the gut microbiota and lessen psoriasis symptoms. Prebiotics encourage the development and activity of beneficial gut bacteria, whereas probiotics are living microorganisms that give health advantages when ingested in sufficient proportions [89]. So, there is increasing evidence that implies that dietary factors may influence the gut microbiota and play a part in the development of this disease. A summary of the two rare inflammatory diseases is discussed in Table 2.

#### 1.4.3. Lupus

Lupus, also known as systemic lupus erythematosus (SLE), is an autoimmune condition that can affect different body organs. The development and course of the illness may be influenced by such environmental factors as nutrition and the microbiota in the gut. According to one study, those with SLE had a different gut microbiota composition from people in the general population, with a lower number of helpful bacteria and a higher number of possibly dangerous bacteria [90]. Other research showed altered gut microbiota that might cause an increase in inflammation, which is a major contributor to the onset of SLE [91]. A study on food discovered that a decreased risk of having SLE was linked to a Mediterranean-style diet, and its higher risk was linked with processed foods, saturated fat, and sugar [92]. This is assumed to be because many of the items in this kind of diet have anti-inflammatory and antioxidant qualities [33]. Overall, it is suggested that a good diet and a balanced gut microbiota may be significant factors in lowering the risk of getting lupus and controlling its symptoms, even though the relationship between lupus, food, and gut microbiota is currently being investigated.

### 1.5. Key Transcriptional Factors

Some key transcriptional factors are enlisted below.

#### 1.5.1. Peroxisome Proliferator-Activated Receptors (PPARs)

PPARs are a class of transcription factors that are essential for controlling lipid metabolism and maintaining homeostasis in the body’s energy supply. PPARs are expressed in a variety of cell types in the gut, such as enterocytes and immune cells. The makeup of the gut microbiota and the interactions between hosts and microbes can be affected by the activation of PPARs by dietary lipids or their metabolites. PPARs can affect the makeup and operation of the gut microbiota by regulating the expression of genes related to lipid metabolism, inflammatory response, and absorption of lipids [93,94].

#### 1.5.2. Liver X Receptors (LXRs)

Their main function is to control the metabolism of cholesterol. Oxysterols, which are byproducts of the oxidation of cholesterol, activate them. By controlling the expression of genes involved in lipid metabolism and inflammation, LXRs can have an impact on the microbial flora in the gut. LXRs have the ability to regulate immunological responses, bile acid production, and cholesterol absorption, all of which can indirectly alter the composition and operation of the gut microbiota [95,96].

#### 1.5.3. Farnesoid X Receptor (FXR)

Nuclear receptors, such as FXR, are mainly found in the liver and gut. It acts as a sensor for bile acids, which are produced in the liver from cholesterol and are essential for the digestion and absorption of lipids. Bile acid production, transport, and metabolism are all regulated by bile acid activation of the FXR. Through these processes, FXR can modify the composition of bile acids, which, in turn, influences the development and activity of certain gut bacteria [97,98].

#### 1.5.4. Intestinal Krüppel-Like Factors (KLFs)

Intestinal epithelium expresses a family of transcription factors known as KLFs. They have a role in controlling lipid metabolism, cell division, and barrier function, among other aspects of intestinal physiology. KLFs could control the expression of genes that are involved in lipid metabolism and absorption, as well as the synthesis of antimicrobial peptides. KLFs can influence the makeup of the gut microbiota and help maintain intestinal homeostasis by controlling these processes [99].

### 1.6. Molecular Mechanism

#### 1.6.1. Regulation of Lipid Metabolism Genes

In response to a diet high in lipids, transcription factors can directly control the expression of genes involved in lipid metabolism. For instance, dietary lipids or their metabolites can activate peroxisome proliferator-activated receptors (PPARs), such as PPAR and PPRE. Peroxisome proliferator response elements (PPREs), which are unique DNA sequences found in the promoter regions of target genes involved in lipid metabolism, can then bind to these activated PPARs. PPARs can influence lipid metabolism in the gut by binding to PPREs and increasing the expression of genes that code for the enzymes involved in fatty acid oxidation, lipid transport, and adipogenesis [100].

#### 1.6.2. Modulation of Inflammatory Responses

The expression of genes involved in inflammatory reactions can be controlled by transcription factors, which may influence the microbial flora in the gut. For instance, the transcription factor nuclear factor-kappa B (NF-B) is essential for inflammation. Several things, notably lipopolysaccharides (LPS) produced by gut bacteria, can activate it. NF-B is a nuclear protein that moves to the nucleus after activation and binds to B sites in the promoters of target genes that are involved in immunological and inflammatory responses. The makeup and activity of the gut microbiota can be impacted by the generation of pro-inflammatory cytokines and chemokines, which can result from NF-B activation [101].

#### 1.6.3. Control of Bile Acid Homeostasis

Bile acids, which the liver produces from cholesterol, are essential for the digestion and absorption of lipids. Bile acid homeostasis is regulated by transcription factors, such as farnesoid X receptor (FXR) and liver X receptor (LXR). Bile acids activate the FXR, which can then bind to FXREs in the promoters of target genes involved in the production, transport, and metabolism of bile acids. FXR can affect the composition and metabolism of bile acids by controlling the expression of these genes, which, in turn, can affect the microbial flora in the gut [102]. The expression of genes involved in bile acid metabolism and cholesterol homeostasis can also be modified by LXRs, thereby affecting the gut microbiota [103].

#### 1.6.4. Crosstalk with Intestinal Epithelial Cells

Transcription factors can also control the interactions between intestinal epithelial cells and the gut microbiota. The intestinal epithelium, for instance, contains a variety of transcription factors, such as Krüppel-like factors (KLFs), which are important in controlling the expression of genes linked to barrier function, cell proliferation, and innate immune responses. KLFs can alter the expression of genes producing tight junction proteins, mucus production, and antimicrobial peptides, affecting host–microbe interactions and the makeup of the gut microbiome [104].

#### 1.6.5. Lacatse Gene

**Figure 5:**
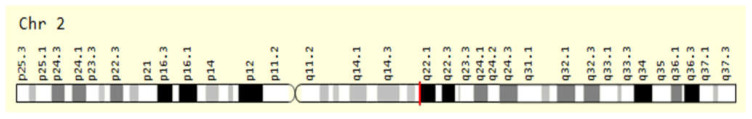
The red line before q22.1 position giving an expression of LCT gene location on chromosome number 2 in humans.

The lactase gene (*LCT*) is responsible for the production of the lactase enzyme, located on chromosome 2 in humans (Figure 5), and contains 50,000 base pairs (Table 3). Variations in the *LCT* gene can lead to such symptoms as abdominal pain, bloating, gas, and diarrhea after consuming dairy products. Some populations around the world, particularly individuals of African, Asian, and Native American descent, have developed genetic variations that allow them to continue producing lactase into adulthood, a trait known as lactase persistence. This is particularly common in 90% of populations of European descent. Research is ongoing, with the hope of better understanding the underlying mechanisms and developing new treatments or interventions for those who struggle with lactose digestion. The enzyme found in the intestinal brush border membrane plays a crucial role in the hydrolysis of lactose, the primary sugar present in mammalian milk. The enzyme is composed of two domains that exhibit catalytic activity toward beta-glucopyranosides and beta-galactopyranosides, with a preference for hydrophilic aglycones present in lactose and cellobiose in one domain and hydrophobic aglycones in phlorizin and glucosylceramides in the other. The enzymatic hydrolysis of lactose by this enzyme results in the production of D-glucose and D-galactose, which are essential for the proper absorption and utilization of these sugars by the body [105].

##### Expression for *LCT* Gene

The expression of the *LCT* gene, which encodes the lactase enzyme, is regulated by several factors. One of the most important regulatory mechanisms is the activity of a region located upstream of the *LCT* gene known as the lactase–phlorizin hydrolase enhancer (LCT-PE). The LCT-PE region contains binding sites for several transcription factors that are necessary for the initiation of gene transcription and subsequent lactase enzyme production. In individuals with lactase persistence, the LCT-PE region remains active throughout their lifetime, allowing them to continue producing lactase into adulthood. In contrast, individuals with lactase non-persistence experience a decrease in *LCT* gene expression and lactase enzyme production as they age, leading to lactose intolerance [106]. In addition to genetic factors, *LCT* gene expression can also be influenced by such environmental factors as diet and gut microbiota. Studies have shown that dietary changes, particularly the consumption of dairy products, can impact *LCT* gene expression and lactase enzyme production. Alterations in gut microbiota cocktail and function have also been linked to changes in *LCT* gene expression and lactase activity, highlighting the multifactorial factors in lactose intolerance [105].

Although the link between gut inflammatory diseases, such as IBD, and the *LCT* gene is not straightforward, research in this area is ongoing, and several factors need to be considered. Firstly, people with IBD may experience lactose intolerance as a secondary effect of their condition. The inflammation in the gut can damage the cells that produce lactase, leading to a reduced ability to digest lactose and causing such gastrointestinal symptoms as bloating, diarrhea, and abdominal pain. Secondly, the *LCT* gene and lactose intolerance may have indirect implications for gut inflammation. Inflammatory bowel diseases involve an abnormal immune response in the gut, and it is possible that dietary factors, including lactose, could influence this response. However, the specific role of lactose or the *LCT* gene in triggering or exacerbating inflammation is not yet well-defined. Lastly, emerging evidence suggests that changes in the gut microbiome, the collection of microorganisms in the digestive tract, may play a role in the development and progression of inflammatory bowel diseases. Some studies have explored whether lactose intolerance, influenced by the *LCT* gene, could contribute to alterations in the gut microbiome that potentially impact inflammation. However, further research is needed to establish a direct link between the *LCT* gene, lactose intolerance, and alterations in the gut microbiome [105,106].

### 1.7. Anti-Inflammatory Effects of Omega-3 Fatty Acids

The anti-inflammatory properties of omega-3 fatty acids, particularly eicosapentaenoic acid (EPA) and docosahexaenoic acid (DHA), have been well-investigated [107]. The following are some of the main ways that omega-3 fatty acids reduce inflammation.

#### 1.7.1. Inhibition of Pro-Inflammatory Mediators

The production of pro-inflammatory mediators, including cytokines, chemokines, and eicosanoids, can be decreased by omega-3 fatty acids. Inflammatory eicosanoids, including prostaglandins and leukotrienes, are produced by the enzymes cyclooxygenase (COX) and lipoxygenase (LOX), which are competitively inhibited by EPA and DHA. Omega-3 fatty acids can aid in decreasing inflammation by lowering the production of these pro-inflammatory mediators [108].

#### 1.7.2. Modulation of Inflammatory Signaling Pathways

Different inflammatory signaling pathways can be disrupted by omega-3 fatty acids. They have the ability to prevent the transcription factor nuclear factor-kappa B (NF-B), which is essential for the start of inflammatory reactions, from becoming activated. In addition, signal transducer and activator of transcription (STAT) proteins and other mitogen-activated protein kinases (MAPKs) can both be inhibited by EPA and DHA [109].

#### 1.7.3. Resolution of Inflammation

When inflammation is under control, omega-3 fatty acids help the body transition from a pro-inflammatory to an anti-inflammatory and pro-resolving state. Resolvins, protectins, and maresins are specialized pro-resolving lipid mediators (SPMs) that actively reduce inflammation and encourage tissue healing when they are stimulated to be produced [110].

#### 1.7.4. Regulation of Immune Cell Function

Various immune cells involved in the inflammatory response, such as macrophages, monocytes, neutrophils, and lymphocytes, can have their functions affected by omega-3 fatty acids. They can influence immune cells’ generation of inflammatory cytokines, improve phagocytosis and the removal of cellular waste, and encourage macrophages to change into an anti-inflammatory phenotype [111,112,113].

#### 1.7.5. Preservation of Cellular Membrane Integrity

Omega-3 fatty acids have an impact on the fluidity and integrity of these membranes. They can control the synthesis and release of inflammatory mediators from immune cells by integrating into cell membranes. Additionally, they are antioxidants and can shield cells from inflammation brought on by oxidative stress [112]. Omega-3 fatty acids have anti-inflammatory properties, which may help treat a variety of inflammatory illnesses, including inflammatory bowel disease, rheumatoid arthritis, cardiovascular disease, and neuroinflammatory disorders. The effectiveness of omega-3 fatty 1.7.acids can change based on the specific ailment, dose, and individual variability, so it is vital to keep that in mind.

## 2. Conclusions

In almost all the diseases that are discussed above, it is concluded that a diet high in fruits, vegetables, whole grains, and lean protein sources has been associated with reduced inflammation and improved immune function as it promotes healthy microbiota of the gut. On the other hand, a Western diet has been linked to increased inflammation and immune dysfunction, thus decreasing the activity and growth of gut microbiota. Inflammatory bowel diseases are complex conditions with multiple factors involved, including genetic predisposition, environmental triggers, and dysregulation of the immune system. Therefore, personalized advice and guidance from a healthcare professional or gastroenterologist are recommended for those with gut inflammatory diseases and potential implications of the *LCT* gene. Further research is needed to fully understand the role of the transcriptional factors and of the *LCT* gene in the development and progression of IBD, and the underlying mechanisms need to be elucidated to develop effective therapies for this debilitating condition. It is essential to note that lactose intolerance is not considered a primary risk factor for developing IBD.

## Figures and Tables

**Table 1:** Summary of the common inflammatory diseases and gut microbiota with reference to diet.

Common Inflammatory Diseases	Beneficial Diet	Harmful Diet	Changes in Gut Microbial Flora due to Harmful Diet	References
**PMR**	Vitamin D, plant-based diet, lean proteins	Processed foods, refined sugars, fats	Increase in Prevotella, Bacteroides, Ruminococcus sp.	Muratore et al., 2015 [24] Zhao et al., 2019 [25]
**SMA**	Calcium, dietary fibers, probiotics	Processed foods	Increase in harmful bacteria.	Zhou et al., 2022 [32] Brzozowski et al., 2016 [33]
**Vasculitis**	Calcium, broccoli, yogurt, skimmed milk, plant-based diet, beta-glucan	Food additives, fried foods, non-digestible carbohydrates	Increase in bacteroides	Lunardi et al., 1992 [35] Sato et al., 2017 [38] Snelson et al., 2023 [40]
**Sarcopenia**	Proteins, vitamin D, omega 3 fatty acids, fruits, vegetables	Western-style foods, including fats and processed sugars and foods	Increase in harmful bacteria.	Smith et al., 2015 [42] Beaudart et al., 2016 [43] Morley et al., 2020 [46]
**Cirrhosis**	Polyunsaturated fats, dietary fibres, whole grains, omega-3 foods (fish, olive oil)	Saturated and monounsaturated fats, high intake of processed and red meat, sugars, and refined carbohydrates	Increase in harmful gut bacteria	Lee et al., 2016 [47] Han et al., 2017 [49] De la Fuente et al., 2020 [52]
**Cancer**	Vitamin D, omega-3 fatty acids, plant-based diet	High intake of processed and red meat	Increase in inflammation-causing bacteria	Fung et al., 2015 [53] Harvie and Howell, 2018 [54] Larsson and Wolk, 2018 [55]; Norris and Dennis, 2019 [56]
**Fibromyalgia**	Low FODMAP diet, fruits, vegetables, lean proteins, whole grains	Processed foods and carbohydrates	Increase in inflammation-causing flora	Pedersen et al., 2017 [59] Bagis et al., 2015 [57]; Castro et al., 2019 [58]

**Table 2:** Summary of the rare inflammatory diseases and gut microbiota with reference to diet.

Rare Inflammatory Diseases	Beneficial Diet	Harmful Diet	Changes in Gut Microbial Flora due to Harmful Diet	References
**Sarcoidosis**	Omega-3 FA, antioxidant fruits and vegetables that are rich in antioxidants	Saturated and trans fats, refined carbohydrates, and processed foods	Altered flora; increase in harmful gut bacteria	Zhuang et al., 2019 [62] Cheng et al., 2020 [60] Gupta et al., 2019 [63]
**Psoriasis**	Fruits, vegetables, fibers, probiotics, and prebiotics	Fats and sugars	Harmful bacterial flora in the gut	Kim et al., 2019 [68] Navarro-López, et al., 2018 [69] Shen, et al., 2017 [70]

**Table 3:** *LCT* gene location and its surrounding in chromosome number 2.

No.	Chr	Strand	Gene	Category	Enhancer	EntrezGeneId
33,471	chr2:135,783,531-135,783,680				GH02J13578	
33,472	chr2:135,786,471-135,787,840				GH02J13578	
33,473	chr2:135,787,850-135,837,184	−	LCT	Protein Coding		3938
33,474	chr2:135,791,413-135,793,045				GH02J13579	
33,475	chr2:135,794,362-135,794,983	+	HSALNG0019075	RNA Gene		
33,476	chr2:135,794,396-135,795,460				GH02J13579	
33,477	chr2:135,797,202-135,798,800				GH02J13579	
33,478	chr2:135,804,934-135,828,172	+	HSALNG0019076	RNA Gene		
33,479	chr2:135,806,054-135,806,133	−	RF02000-004	RNA Gene		
33,480	chr2:135,810,169-135,810,279	−	ENSG00000200664	RNA Gene		

## Data Availability

Not applicable.
